# The use of erlotinib in daily practice: a study on adherence and patients' experiences

**DOI:** 10.1186/1471-2407-11-284

**Published:** 2011-07-01

**Authors:** Lonneke Timmers, Christel CLM Boons, Dirk Mangnus, Josee E Moes, Eleonora L Swart, Epie Boven, Egbert F Smit, Jacqueline G Hugtenburg

**Affiliations:** 1 Department of Clinical Pharmacology and Pharmacy, VU University Medical Center, De Boelelaan 1117, 1081 HV Amsterdam, the Netherlands; 2 Department of Pulmonary Diseases, VU University Medical Center, Amsterdam, the Netherlands; 3 Department of Medical Oncology, VU University Medical Center, Amsterdam, the Netherlands

## Abstract

**Background:**

Adherence to pharmacological therapy is a complex and multi-factorial issue that can substantially alter the outcome of treatment. It has been shown that cancer patients, especially when using long-term medication, have similar adherence rates to those of patients with other diseases. The consequences of poor adherence are poor health outcomes and increased health care costs. Only few studies have focused on the use of oral anticancer agents in daily practice. Information about the reasons for non-adherence is essential for the development of interventions that may increase adherence. This paper presents the CAPER-erlotinib protocol, which is designed to study the relationship between adherence to erlotinib and both the plasma concentration and side-effects in patients with NSCLC. Further, the relationships between patient characteristics, disease characteristics, side-effects, quality of life, patient beliefs and attitude towards disease and medication, dose adjustments, reasons for discontinuation and plasma concentration of erlotinib will be explored.

**Methods/Design:**

In this prospective observational cohort study 65 NSCLC patients of 18 years or older starting treatment with erlotinib will be followed for a period up to 16 weeks. The main study parameters are adherence, the plasma concentration of erlotinib and the number and grade of side-effects. At baseline and on erlotinib treatment in weeks 3-4, 8-9, 12 and 15-16, patients will be asked to fill out a questionnaire. In weeks 3-4, 8-9 and 15-16 blood samples are collected, which will be analysed for plasma concentration of erlotinib. Adherence will be measured using a medication event monitoring system.

**Discussion:**

The present study aims to get more insight into patients' experiences with the use of erlotinib in daily practice and the various aspects that govern adherence. We hypothesize that side-effects play an important role in the way patients use erlotinib. We expect that the present study will provide valuable knowledge which will be useful for health care professionals to develop interventions to support patients. This approach will improve the adherence and persistence with the use of erlotinib in order to derive optimal benefit from the medication.

**Trial Registration:**

NTR1830

## Background

In the pharmacological treatment of cancer intravenous (IV) therapy has played an important role. Since the last decade a growing number of oral substances has been introduced in cancer treatment. Most patients prefer oral use of anticancer agents as long as it does not compromise the outcome of treatment [1,2]. In addition, the overall costs of oral treatment are often lower than those of IV therapy [3-5]. However, with the use of oral medication at home in daily practice the issue of adherence needs to be considered.

### Adherence

Adherence to oral pharmacological therapy is a complex and multifactorial issue that can substantially alter the outcome of therapy [6,7]. Adherence (synonymous with compliance) has recently been defined by the International Society for Pharmacoeconomics and Outcome Research (ISPOR) as the extent to which a patient acts in accordance with the prescribed interval and dose of a dosing regimen [8]. A patient is optimally adherent if no doses are missed, no extra doses are taken, and no doses are taken in the wrong quantity or at the wrong time. Adherence is measured over a period of time and reported as the adherence rate, which is the percentage of dose taken in relation to what was prescribed [9]. There are several methods to measure adherence, including self reports, pill counts, electronic monitoring systems, analyses of pharmacy dispensing databases or assessment of blood or urine samples. There is no golden standard measurement and all methods have limitations [9,10]. The major limitation of measuring adherence is the so called Hawthorne effect: the measuring of adherence itself influences the adherence because the awareness of patients that adherence is being measured may influence their behaviour. Adherence rates for many chronic drug therapies have been shown to range between 35-70% [11]. The consequences of poor adherence are poor health outcomes and increased health care costs [6].

### Adherence in oncology

Cancer patients are generally thought to have higher adherence rates than other patients because they are highly motivated by the gravity of their disease [12,13]. However, it has been shown that cancer patients have similar adherence rates to those of patients with other diseases [9,14,15]. Treatment duration plays a role in the adherence to the regimen. When the medication is continued over a longer period of time, patients become less adherent [16].

In oncology adherence has been studied mainly in two subpopulations, both using long-term medication. In the first population, breast cancer patients on adjuvant hormonal therapy like tamoxifen the adherence rate has been subject of several studies [9]. The reported adherence rates range from 50% to 98% [13,16,17]. Several studies concerning adherence to oral medication have been published in the second subpopulation, patients with Chronic Myeloid Leukemia (CML) [15,18,19]. Non adherence appear to be associated with poorer response to imatinib [15,18]. Noens et al have shown that patients with suboptimal response had higher percentages of imatinib not taken (23%) than those with optimal response (7%). Marin et al have demonstrated in CML that there was a strong correlation between adherence rate and response. Adherence was the only critical factor for achieving molecular response.

Another frequently overlooked problem is over-adherence. This may be more an issue in oncology patients than in other patients and may, in the case of oral anticancer agents, lead to substantially increased toxicity [10]. In the study by Nilsson et al. [14], 30% had an oversupply of their medication.

Oncologists may not always consider the issue of adherence. Yet, suboptimal adherence may prove to be the greatest barrier to the effective use of new oral agents [9,18].

### Causes of non-adherence

Several factors have shown to be associated with non-adherence. These include patient variables like demographic factors, patients beliefs, disease variables, various aspects of treatment regimes, side- effects and quality of life.

The common sense model of self-regulation (CSM) developed by Leventhal et al [20] is a commonly used theoretical model to understand patients perceptions of illness. According to the CSM peoples' perception of and beliefs about their illness is an important factor in their reactions and behaviour to health threats. Illness perceptions can easily be measured with the Brief Illness Perceptions Questionnaire (Brief IPQ) [21].

Another factor which may influence adherence is patients beliefs about the medication. These representations are usually determined with the Beliefs about Medicine Questionnaire (BMQ) [22]. Associations between adherence and BMQ scores have been shown[23-27].

Few studies have focused on patients' reasons for not adhering to oral anticancer agents. Eliasson et al [28] have reported that finding ways to deal with the side-effects leads to better adherence in patients with CML. In clinical trials adverse events are generally reported by clinicians, e.g. the National Cancer Institute's Common Terminology Criteria for Adverse Events (CTCAE). Basch et al have shown the value of patients' reported adverse symptom events in cancer patients, which better reflect their daily health status [29,30].

### Erlotinib

Erlotinib (Tarceva^®^) is an oral anticancer agent, which is registered for the second- and third-line treatment of NSCLC since 2006 [31] Erlotinib is a tyrosine kinase inhibitor (TKI) of epidermal growth factor receptor (EGFR-TKI).

Diarrhea and skin rash are the most common side-effects of erlotinib. The incidence and severity of diarrhea seems to be related to the schedule and dose of erlotinib [32]. Rudin et al [33] have not observed a relationship between the Area Under the Curve (AUC) concentration and diarrhea. However, they did find a relationship between trough plasma concentration (C_trough_) of erlotinib and skin rash. Hidalgo et al [32] also detected a relationship between AUC and skin rash. Interestingly, patients who experienced skin rash had significantly longer survival [34,35].

### Pharmacokinetics

Hidalgo et al [32] have suggested that an erlotinib plasma concentration of 0.5 μg/ml would be sufficient for relevant antiproliferative activity. Peak concentrations (Cmax) of erlotinib of 1.737 ± 0.777 μg/ml are reached 2.44 ± 1.94 hour (Tmax) after administration of 150 mg a day. Erlotinib has a plasma volume of 233 L and is for 95% bound to plasma proteins like albumine and α1-acid-glycoprotein. The plasma elimination half-life is approximately 36 hours [36]. Erlotinib is mainly metabolized by the liver (90%), only a small part is excreted by the kidneys (9%). It is primarily metabolized by cytochrome P450 3A4 (CYP3A4) and in a lesser extent by CYP1A2, It is a substrate of the multi-drug transporter ATP-binding cassette transporter ABCG2 [37].

Several drug interactions, smoking and food may influence plasma concentrations of erlotinib. Drugs that increase the pH of the upper gastrointestinal tract may alter the solubility of erlotinib, thereby reducing its bioavailability. Inhibitors or inducers of CYP3A4 and CYP1A2 influence the metabolism of erlotinib, which may result in increased or decreased plasma concentrations [31] Smoking results in a significantly lower plasma concentration [38]. When erlotinib is taken together with food the bioavailability of erlotinib increases almost two-fold [39]. It is therefore advised by the manufacturer to take erlotinib at least one hour before or two hours after a meal [31]. Serum total bilirubin, serum α1-acid glycoprotein, serum albumin, creatinine clearance and gender are significant co-variables that explain interindividual variability for clearance [36].

### Objectives

The primary objective is to get insight into the relationship between adherence to erlotinib and the plasma concentration of erlotinib and the relationship between adherence and side-effects in patients with NSCLC. Furthermore, the relationships between patient characteristics, disease characteristics, side-effects, quality of life, patient beliefs and attitude towards disease and medication, adherence, dose adjustments and plasma concentration of erlotinib in patients with NSCLC will be explored.

## Methods/Design

### Study design

A multicenter, prospective observational cohort study in which patients starting treatment with erlotinib will be followed for a period up to 16 weeks. At baseline and in weeks 3-4, 8-9, 12 and 15-16, patients will be asked to fill out a questionnaire. Furthermore, in weeks 3-4, 8-9 and 15-16, blood samples are collected, which will be analysed for plasma concentration of erlotinib. Adherence will be measured using a medication event monitoring system (MEMS). Information from the physician will be retrieved from the patient treatment file.

### Ethics

The protocol was approved by the Medical Ethics review board of the VU University Medical Center, Amsterdam, The Netherlands.

### Recruitment and consent

Patients will be recruited by the Departments of Pulmonalogy from twelve hospitals in the Netherlands. After the patient and doctor have decided on treatment with erlotinib, patients will be informed about the study and will receive written information to take home. Within two days the researcher will contact the patient by phone to further inform the patient of the study and to ask for his/her consent. If the patient decides to participate in the study, he or she will sign the informed consentform.

### Inclusion criteria

Patients with advanced NSCLC under treatment in one of the participating hospitals starting with the use of erlotinib can be included. Patients younger than 18 years and those with insufficient knowledge of the Dutch language will be excluded. According to the sample size calculation (see Statistics) a number of 65 patients will be enrolled.

### Measurements

Several methods are used to determine the variables. Most information is collected directly from patients: they will fill out questionnaires, will use an electronic monitoring pill-container and will donate blood samples. Furthermore, medical information will be retrieved from the patients' file.

#### Questionnaires

The patients will be asked to fill out five questionnaires spread over 16 weeks. The first questionnaire (t = 0) contains questions on patient characteristics, smoking, co-medication, side-effects, quality of life, and patients beliefs and attitude towards disease and medication. In weeks 3-4, 8-9 and 15-16 patients will be asked to fill out an elaborate questionnaire containing questions determining the adherence behaviour, side-effects, dose adjustment, co-medication, quality of life, and patients beliefs and attitude towards disease and medication. In week 12 patients are asked to fill out a short questionnaire containing only questions determining the adherence behaviour, side effects, and dose adjustment. Discontinuation and reasons for discontinuation will be asked in a short questionnaire when a patient stops the erlotinib treatment prematurely.

The questionnaires contain the following items:

1. MARS (Medication Adherence Report Scale) [40,41]. This questionnaire contains statements about adherence behaviour.

2. Nature and grade of the side-effects in a five-point scale.

3. Quality of life measured with SF-12 Health Survey [42,43]. The SF-12 is a short version of the SF-36 and is a validated method to measure quality of life.

4. Attitude towards disease measured with Brief Illness Perception Questionnaire (Brief IPQ) [21]. The Brief IPQ is a validated method to measure the attitude towards disease.

5. Beliefs and attitude towards medicines measured with Beliefs about Medicines Questionnaire (BMQ) [22]. The BMQ is a validated questionnaire. It is divided in two parts, the BMQ general and the BMQ specific. BMQ general measures the patient's beliefs and attitude towards medicines in general. The BMQ specific is specified for erlotinib.

6. Patient characteristics: date of birth, gender, socio-economic status and smoking (current smoking and never/previous smoking).

7. Dose adjustments by the patient. Dose adjustments introduced by the doctor will be derived from the patient file.

8. Co-medication.

9. Discontinuation and reasons for discontinuation. Discontinuation is also derived from the patient file.

#### Electronic monitoring system

Adherence will be measured using a medication event monitoring system (MEMS) [44]. Electronic monitoring systems use standard pill containers with a small electronic processor in the cap, to record the timing and frequency of bottle openings. Compared to other methods (e.g., assay, self-report, collateral report, prescription refills), electronic monitoring captures more of the dynamics of medication-taking behaviour [45]. In this study the Evalan Real Time Medication Monitoring (RTMM) is used.

#### Patient file

The following items are derived from the patient treatment file.

• Disease characteristics like disease stage and line of treatment.

• Dose adjustments

• Discontinuation and reasons for discontinuation

#### Blood sample and laboratory test

A plasma sample will be collected at visits of the patient at week 3-4, 8-9 and 15-16. This blood sample will be drawn simultaneously with the regular treatment's related blood sampling. At these visits patients are asked when they had their last meal and at what time they took their last erlotinib medication. Patients are supposed to take erlotinib at least one hour before or two hours after a meal [31]. Adherence to the instruction will be taken into account in the analysis of the study results.

Apart from that, the time of the blood sample will be documented. Plasma concentrations of erlotinib will be analyzed by LC-MS/MS. The pharmacokinetic parameters AUC, C_max _and C_through _will be derived using the pharmacokinetic model as described by Lu et al [36]. The concentration ratio (CR) is defined as the measured erlotinib concentration divided by the mean population erlotinib concentration that is predicted by the model. To calculate the AUC, C_max_, C_through _and CR the following covariates are measured and used as input for the model: gender, serum albumin, serum total bilirubin, serum α1-acid glycoprotein, calculated creatinine clearance and smoking status [36]. The calculated AUC, C_max_, C_through _and CR will be compared with the data on adherence from the questionnaires and the electronic monitoring system to study the relationship between adherence and the plasma concentration of erlotinib.

Patients are asked about their co-medication in the questionnaires. The data on co-administration of inhibitors of CYP1A2 and CYP3A4, inductors of CYP3A4 and antacids will be examined as covariables.

### Statistics

The data will be described by means of frequency analysis. Correlation coefficients will be calculated for relationships between adherence, patient characteristics, disease characteristics, side effects, quality of life, patient beliefs and attitude towards disease and medication, dose adjustments and plasma concentration of erlotinib in patients with NSCLC. In particular, the correlation coefficient between plasma concentration and side-effects; the relationship between dose and plasma concentration in daily practice; the influence of different factors on adherence; the influence of different factors on side-effects; the influence of different factors on quality of life; the influence of different factors on dose adjustment and discontinuation. By the use of a multivariate regression analysis the influence of adherence (dependent variable) on plasma concentration and the influence of side-effects (dependent variable) on adherence will be calculated. The dependent variables will be adjusted for potential confounders. For all analyses a two-tailed significance level will be 0.05, all p values below this level will be considered statistically significant. The statistical analyses will be performed with SPSS 15.0 (SPSS Inc. Chicago, Illinois, USA).

## Discussion

The present study aims to get more insight into patients' experiences with the use of erlotinib in daily practice. We expect variability in the adherence in the general oncology population. This may be complicated by the inter-patient variability in the pharmacokinetics. We hypothesize that side-effects play an important role in the way patients use erlotinib. Therefore, the relationship between self-reported side-effects and adherence is also defined as a main objective. To get more insight into other factors related to adherence and other aspects of the use at home, the effects of determinants are studied in an explorative manner.

We expect that the present study will provide valuable knowledge on patients' experiences with the use of erlotinib. This knowledge will be useful for health care professionals to develop specific interventions to support patients thus improving the adherence and persistence with the use of erlotinib in order to derive optimal benefit from the medication.

## Competing interests

The authors received an unrestricted grant from Roche Netherlands. The authors declare that they have no competing interests.

## Authors' contributions

JH and LT designed the study and obtained funding. ELS, EFS, EB and CB contributed to the study protocol. DM and JM contributed to the pharmacokinetics section. CB and LT will implement the multicenter protocol and collect the data. All authors read and approved the final manuscript.

## Pre-publication history

The pre-publication history for this paper can be accessed here:

http://www.biomedcentral.com/1471-2407/11/284/prepub
